# Ultrasound in evaluation of post-interventional femoral vein obstruction: a case report

**DOI:** 10.1186/1476-7120-7-14

**Published:** 2009-03-26

**Authors:** Mai Tone Lønnebakken, Eva Gerdts, Jan Wirsching, Ole Martin Pedersen

**Affiliations:** 1 Department of Heart Disease, Haukeland University Hospital, Bergen, Norway; 2 Institute of Medicine, University of Bergen, Bergen, Norway; 3 Department of Radiology, Haukeland University Hospital, Bergen, Norway

## Abstract

Ultrasound is the preferred imaging modality in diagnosis of vascular complications following cardiac catheterization and intervention. In some cases, however, bleeding surrounding the femoral vessels, may severely distort the color Doppler images, making detection of venous complications especially difficult. This report refers to such a case where post-catheterization haematoma was suspected to cause an obstruction of the femoral vein. Spectral Doppler recordings of blood flow in the common femoral vein, up-stream, distal to the hemorrhagic area, confirmed the diagnosis of obstruction by demonstrating changes in the venous flow pattern in the common femoral vein, consistent with venous hypertension. Due to the poor quality of the ultrasound images, the exact cause of the obstruction had to be established by another imaging modality, not affected by haemorrhages. CT showed that the common femoral vein was compressed at the puncture site by surrounding haemorrhages. Thus, when bleeding due to cardiac catheterization is associated with possible venous obstruction and findings by color Doppler are equivocal due to degradation of the color-Doppler image, detection of venous hypertension by spectral Doppler, performed distal to the bleeding area, strongly supports the presence of venous obstruction where the exact cause may be established by CT.

## Background

Cardiac catheterization, using the femoral approach, is not infrequently associated with local vascular complications occurring in 1.5–9.0% of cases [1], especially arterial haemorrhage, but may also involve the common femoral vein (CFV), where venous obstruction may occur. Venous obstruction, which is often partial and relatively short, is rarely mentioned in the literature and most probably remains undiagnosed in many cases [2,3].

Color Doppler is the preferred imaging modality in detection and follow-up of venous obstruction. On plain ultrasound (US) images, major veins appear as oval, echo-free structures surrounded by echogenic subcutaneous tissue. Unobstructed veins are completely compressed when moderate pressure is applied to the skin above by the transducer. Unobstructed venous flow, detected by spectral Doppler, is characterized by velocity variations that are synchronous with respiration. When a vein becomes obstructed by a thrombus it becomes incompressible. Simultaneously there is a rise in the vein pressure distal to the obstruction. When the pressure exceeds the peak pressure level in the abdominal veins the characteristic phasic variations in venous flow velocity disappear [4-6].

Post-interventional bleeding into the tissues surrounding the femoral vessels may cause increased scattering of the US echoes leading to distortion of the US images. In addition the bleeding tends to reduce echoes coming from the vein walls, causing the vein echo to blend in with surrounding hypoechogenic haemorrhages and thus no longer be recognized as a separate entity. In these cases, therefore, color Doppler imaging alone may not be fully adequate in the assessment of a possible venous obstruction.

## Case presentation

A 54-years-old man was admitted with a non-ST-elevation myocardial infarction. He was treated with standard combined antiplatelet therapy (aspirin and clopidogrel) and low-molecular weight heparin. Using a right femoral approach, he underwent percutaneous coronary intervention with stent implantation in a stenotic right coronary artery. During the first hours after the procedure, despite an apparent successful closure of the hole in the arterial wall by a collagen plug device (Angioseal, St Jude Medical) and standard compression according to department procedures, he developed pain in the right groin followed by a small haematoma and slight swelling of the right leg.

**Figure 1:**
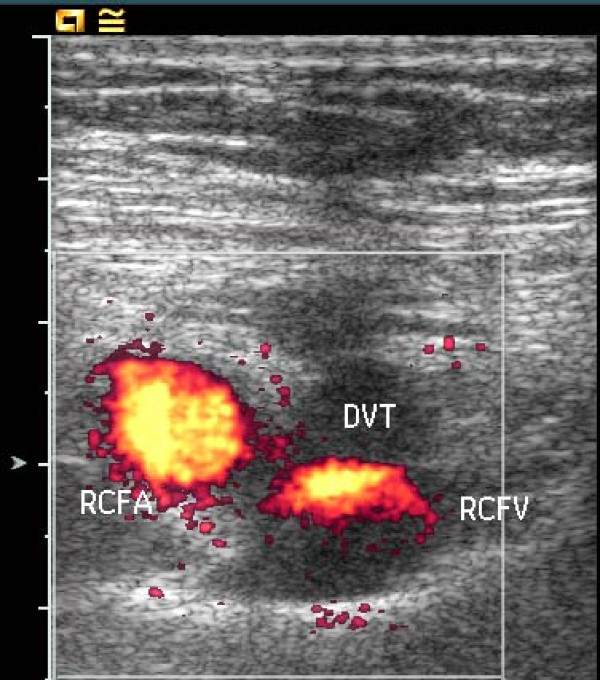
**Short axis Power Doppler image showing the right common femoral artery (RCFA) and the non-compressible right femoral vein (RCFV), surrounded by hypoechogenic tissue consisting of perivascular hematoma, at first suspected to represent deep vein thrombosis (DVT)**.

**Figure 2:**
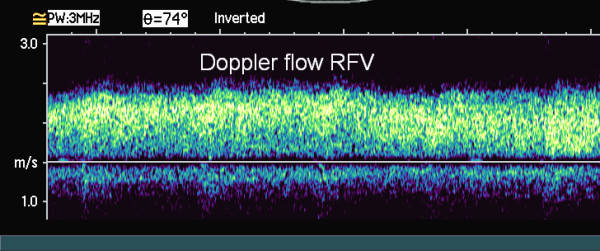
**Spectral Doppler imaging of flow in the right common femoral vein showing persistent high velocity venous flow with loss of normal respiratory synchronous variation consistent with "down-stream" venous obstruction**.

**Figure 3:**
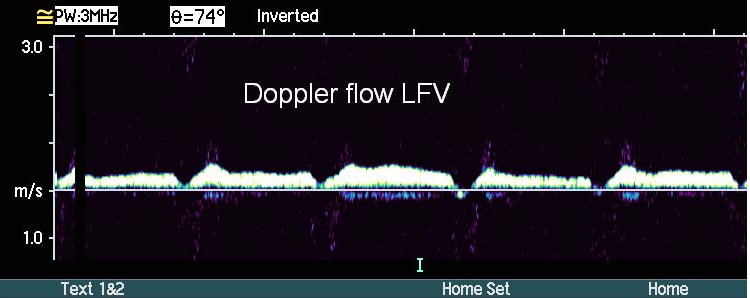
**Spectral Doppler imaging of normal venous flow in the left common femoral vein (LCFV), characterized by normal velocities, that vary synchronous with respiration**.

**Figure 4:**
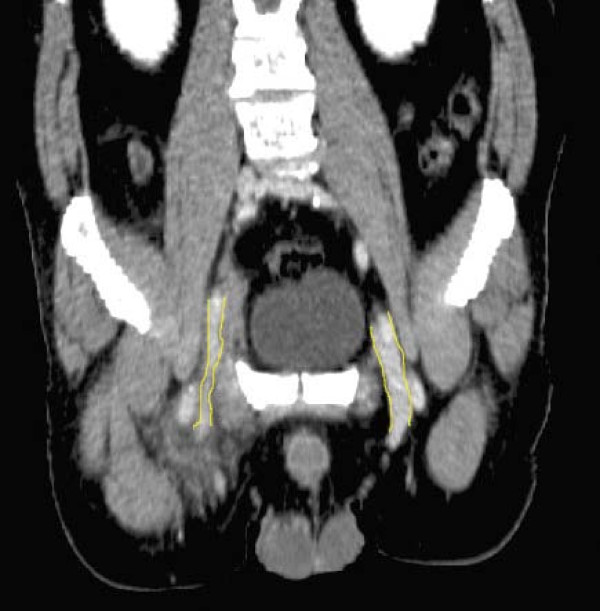
**CT image demonstrating the external compression of the right common femoral vein compared by the normal left common femoral vein**.

US examination of the proximal right CFV, the following day, showed signs of bleeding at the arterial puncture site, which caused considerable degradation of the color-Doppler image due to scattering of the ultrasound. Despite these problems color Doppler demonstrated a 5 cm long cylindric hypo-echogenic structure with a centrally located channel of venous blood flow medial to the CFV (Fig. 1). A possible venous obstruction, due to a partially occluding intraluminal thrombus or to extrinsic compression of the vein lumen by surrounding haematoma, could not be ruled out. Spectral Doppler recordings, obtained away from the puncture site at the distal part of the right CFV, demonstrated unilateral loss of the normal phasic variations in flow velocities, consistent with venous hypertension (Fig. 2, 3). Subsequent assessment of the proximal CFV by CT, being unaffected by haemorrhages, demonstrated partial compression of a 5 cm long vein segment of the CFV at the level of the arterial puncture site, caused by surrounding haematoma (Fig. 4).

## Discussion

Local vascular complications following cardiac catheterizations are not uncommon, the incidence of which are dependent on procedural and patients characteristics [1] and may reach 1.5–9.0%. The arterial complications include hemorrhagic, haematomas, pseudoaneurysms, arteriovenous fistula, acute arterial occlusions, cholesterol emboli, and infections [1]. The incidence of venous complications, especially obstructions where DVT can be potentially life threatening, is seldom mentioned.

This case report demonstrates that, although color Doppler imaging is the preferred modality in diagnosing venous obstruction following cardiac catheterization, bleeding into the surrounding tissues may in some cases cause severe degradation of the color Doppler images and thus may preclude adequate visualization of the CFV. Due to lack of distinct vein wall echoes, the CFV blends in with the surrounding hypoechogenic, thrombus-like haemorrhages, making differentiation between an intravenous thrombus and a perivascular haematoma compressing the vein from the outside virtually impossible [4,7-9].

Our findings show that in these cases spectral-Doppler recordings, performed away from the bleeding area, at the distal end of the CFV, was able to demonstrate venous flow disturbances consistent with venous hypertension supporting the clinical suspicion of venous obstruction down-stream, at the groin.

The problem of inadequate US visualization of the CFV in connection with cardiac catheterizations has been discussed in a recent publication where it was warned against overlooking short partial obstructions of the CFV [10]. In another publication concerning DVT of the CFV following percutaneous cardiac intervention, the authors found DVT of the CFV to be quite common, occurring in as many as 2.4% of patients [3]. DVT was typically located at the arterial puncture site, the involvement of CFV was short, and the obstruction was often partial.

Therefore, in cases where the femoral vessels are insufficiently visualized by color-Doppler due to bleeding at the puncture site, the US operator should use spectral-Doppler recordings to assess venous flow up-stream, at the level of the distal CFV or the proximal part of the superficial femoral vein, looking for signs of venous hypertension secondary to venous obstruction at the groin. Due to inferior quality of color-Doppler images in these cases, CT, which is largely unaffected by bleeding, may be of considerable help in establishing an exact cause of the venous obstruction.

## Consent

Written informed consent was obtained from the patient for publication of this case report and accompanying images.

## Competing interests

The authors declare that they have no competing interests.

## Authors' contributions

MTL carried out the US examination and drafted the manuscript. EG revised the manuscript. JW carried out the interpretation of the CT examination and revised the manuscript. OMP helped in the evaluation of the US examination and drafting of the manuscript.
